# Challenges in Design and Fabrication of Flexible/Stretchable Carbon- and Textile-Based Wearable Sensors for Health Monitoring: A Critical Review

**DOI:** 10.3390/s20143927

**Published:** 2020-07-15

**Authors:** Jae Sang Heo, Md Faruk Hossain, Insoo Kim

**Affiliations:** 1 Department of Medicine, University of Connecticut School of Medicine, Farmington, CT 06030, USA; heo@uchc.edu; 2 Frank Reidy Research Center for Bioelectrics, Old Dominion University, Norfolk, VA 23529, USA; fhossain@odu.edu; 3 Department of Biomedical Engineering, University of Connecticut, Storrs, CT 06269, USA

**Keywords:** carbon-based materials, electronic textiles (e-textile), wearable flexible electronics, flexible/stretchable materials, health monitoring applications

## Abstract

To demonstrate the wearable flexible/stretchable health-monitoring sensor, it is necessary to develop advanced functional materials and fabrication technologies. Among the various developed materials and fabrication processes for wearable sensors, carbon-based materials and textile-based configurations are considered as promising approaches due to their outstanding characteristics such as high conductivity, lightweight, high mechanical properties, wearability, and biocompatibility. Despite these advantages, in order to realize practical wearable applications, electrical and mechanical performances such as sensitivity, stability, and long-term use are still not satisfied. Accordingly, in this review, we describe recent advances in process technologies to fabricate advanced carbon-based materials and textile-based sensors, followed by their applications such as human activity and electrophysiological sensors. Furthermore, we discuss the remaining challenges for both carbon- and textile-based wearable sensors and then suggest effective strategies to realize the wearable sensors in health monitoring.

## 1. Introduction

Recently, wearable health-monitoring sensors have become a part of our daily life. The real-time monitoring of physiological and behavioral data using wearable sensors provided us new culture and information for a healthy lifestyle [1,2,3]. These wearable sensors are commonly attached to the human body or an object and interact with an external or host computer to provide information about the human’s physical and physiological conditions such as activities, stress, sleep, and blood pressure [4,5,6]. The state-of-the-art wearable sensors are light and low-power enough to be embedded in not only smartphones but electronic watches, glasses, and bands that can be easily carried [7,8,9]. Furthermore, wearable sensors are expected to be developed in the form of clothing, human body and skin attachment, or implanted devices in the close future. These new form factor sensors will greatly improve portability, sensitivity, selectivity, and signal-to-noise ratio [1,3]. However, current wearable health-monitoring sensors have technical limitations in advancing into these new form factors. Conventional rigid-type sensor devices have been used in a variety of applications of wearable electronics because they can be easily manufactured by conventional processes. The existing rigid structure shows high electrical performance, but it is difficult to obtain accurate physiological signals because it is not easy to make strong contact with the human body. In addition, there is a problem that electrical performance is deteriorated when deformed by bending, stretching, or twisting [10,11,12,13]. This indicates that the wearable sensor device requires high deformability that can maintain conformal contact with the skin and mechanical stability for long-term use. In order to address this issue, many studies focus on the development of flexible and conformal electronics such as electronic-skin (e-skin) for human activity and physiological signal monitoring systems [14,15,16]. In particular, flexible/stretchable electronics have shown improvement in the sensitivity and accuracy of the sensor when detecting various electrical, mechanical, and biological signals due to their outstanding deformable and conformal characteristics, providing great potential for wearable applications in health monitoring [17,18,19]. Among various applications of flexible/stretchable electronics, detecting mechanical properties such as strain, pressure, and flexion is of great importance for obtaining accurate movement or activity measurements from the human body. Monitoring physiological signals such as electrocardiogram (ECG), electromyogram (EMG), and electroencephalogram (EEG) with a form of wearable sensors could be one of the key applications of flexible/stretchable electronics [20,21,22,23,24].

Carbon- and textile-based electronics for human motion detection and human health monitoring appear to be highly suitable due to their outstanding properties in terms of flexibility and/or strechability. Among various flexible or stretchable materials, carbon-based materials such as carbon nanotubes (CNTs), graphene-based materials, and carbon black (CB) have been extensively used in wearable sensors because of their excellent electrical conductivity, low-cost and potentials for mass production, high chemical and thermal stability, inherent flexibility, and ease of chemical functionalization. Several research groups have reported carbon-based wearable sensors with strain or pressure sensing, with significant improvements in sensitivity and response time using various micro-structural designs, highly conductive and elastic conductors (composites), or new fabrication technologies [14,20,25].

In addition to carbon-based materials, electronic textiles (e-textiles) have attracted attention in industry and academia and can be applied to wearable health monitoring sensors, electronic prostheses, and e-skins due to their unique properties such as lightweight, breathability, comfortability, and wearability [26,27]. Various materials such as organic and mechanically stable inorganic materials can also be embedded in flexible/stretchable e-textiles as active sensing materials, broadening their application ranges and applying various fabrication technologies [28,29].

**Figure 1:**
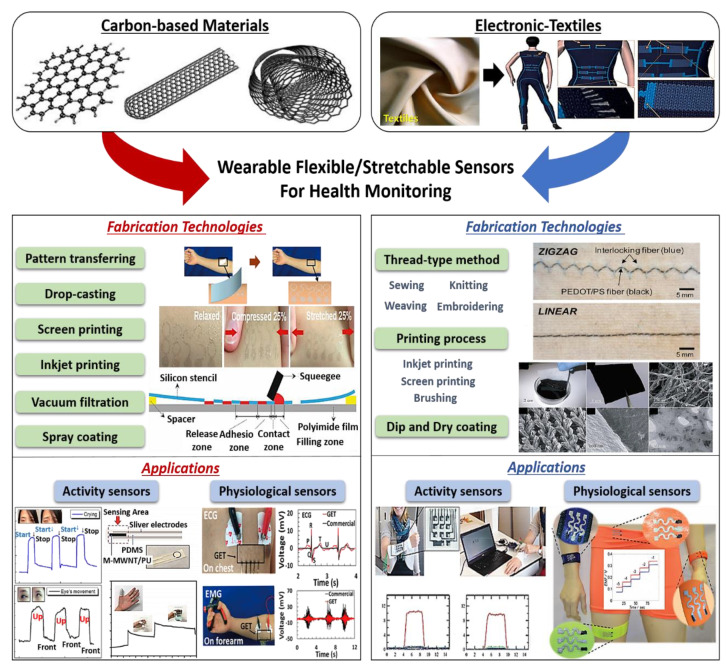
Schematic of effective approaches for wearable flexible/stretchable sensor applications in health monitoring: (i) Carbon-based materials (reproduced with permission [25] copyright 2008, American Chemical Society) and (ii) Electronic textiles (e-textiles) (reproduced with permission [21] copyright 2014, Royal Society of Chemistry). Fabrication technologies; screen printing (reproduced with permission [31] Copyright 2015, Wiley-VCH), pattern transferring (reproduced with permission [30] copyright 2017, American Chemical Society), dipping and drying (reproduced with permission [32] copyright 2010, American Chemical Society), weaving (Reproduced with permission [33] copyright 2017, American Chemical Society). Applications using carbon-based materials; activity sensors: strain sensor (reproduced with permission [34] copyright 2015, American Chemical Society) and pressure sensor (reproduced with permission [35] copyright 2018, MDPI AG). Physiologic sensors: electrocardiogram (ECG) and electromyogram (EMG) sensor (reproduced with permission [30] copyright 2017, American Chemical Society). Applications using e-textiles; activity sensors: capacitive-type pressure sensor (reproduced with permission [36] copyright 2016, Wiley-VCH). Physiologic sensors: a textile-based wearable multi-ion potentiometric sensor (reproduced with permission [37] copyright 2017, Wiley-VCH).

Two strategies, including carbon-based materials and e-textiles, are great candidates in wearable sensors associated with health monitoring, but there are still significant challenges for practical wearable applications in terms of functional materials properties and fabrication technologies that enable accurately detection of various human activities as well as physiological signals [30,31,32,33]. In this article, we focus on recent trends in developing flexible/stretchable electronics for wearable sensors in health monitoring. In particular, two strategies, including carbon-based sensors and e-textiles, are discussed by summarizing advanced functional materials and fabrication technologies and describing their application in wearable applications, as shown in Figure 1 [34,35,36,37]. In addition to the challenges to wearable sensors based on carbon and e-textiles, we review various effective research that created flexible and stretchable electronics made by carbon materials or textile configurations. We believe this review will lead to a discussion of effective strategies for next-generation wearable health monitoring sensors.

## 2. Carbon-Based Wearable Sensors

In general, demonstrating wearable sensors capable of detecting various biometric signals and human activity requires functional materials, such as carbon-based materials (graphene, CNTs, and CB), nanomaterials (nanowires (NWs), and nanoparticles (NPs), that show high electrical properties, high deformability, and biocompatibility with human skin or body [14,15]. Especially, among these materials, carbon-based materials are best suited for applications to flexible and stretchable sensor devices not only because of their outstanding mechanical and electrical performances, low-cost, high chemical/thermal stability, and human compatibility, but also the ease of functionalization using inexpensive and easy methods (purification, doping, oxidation, and reduction reactions) [16,26].

Thus, this section mainly discusses fabrication technologies and applications for carbon-based flexible and stretchable sensors. In particular, we review experimental methods and electrical performances of carbon-based devices for activity, electrophysiological, and physiochemical sensors, including comprehensive fabrication technologies. We also discuss the remaining challenges in this field of study and effective strategies for successfully demonstrating carbon-based wearable sensors.

### 2.1. Fabrication Technologies for Carbon-Based Wearable Sensors

For efficient production of wearable devices, manufacturing technology should be easy and inexpensive. In this regard, carbon-based materials have been considered as appropriate sources due to their compatibility with a variety of solvents, solution-processability, and easy fabrication. There are numerous methods of manufacturing carbon-based materials for application in various electronic devices. However, not all of them are acceptable for demonstrating flexible/stretchable wearable sensors. In this section, we review the acceptable fabrication processes such as pattern transfer, spray coating, drop casting, and printing processes that can be used in carbon-based wearable sensor devices, and discuss their challenges.

#### 2.1.1. Pattern Transferring Process

**Figure 2:**
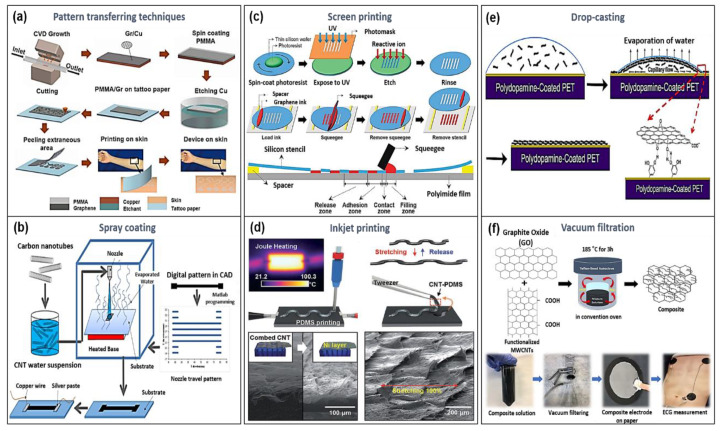
Fabrication technologies for carbon-based wearable sensors. Pattern transferring process; (**a**) transferred chemical vapor deposition (CVD) graphene on the flexible substrate with adhesive polymer and dry patterning process (reproduced with permission [30] copyright 2017, American Chemical Society). Spray coating; (**b**) spray deposition modeling (SDM) with a digital x–y plotter combined with a heated substrate for demonstrating carbon nanotube (CNT)-based sensors (reproduced with permission [44] copyright 2017, Elsevier). Screen and inkjet printing; (**c**) graphene with high-resolution patterning by using screen printing with the thinned silicon-based stencil (reproduced with permission [31] copyright 2015, Wiley-VCH). (**d**) A stretchable CNT-poly(dimethylsiloxane) (PDMS) electrode using both inkjet printing and viscous PDMS controlled by Joule heating (reproduced with permission [52] Copyright 2017, Wiley-VCH). Drop-casting and vacuum filtration; (**e**) reduced graphene oxide (rGO) film deposition onto a functionalized polyethylene terephthalate (PET) substrate using drop-casting process (reproduced with permission [58] copyright 2014, Elsevier). (**f**) Paper-based hybrid chemically modified graphene and carboxylic groups-functionalized short multi-walled carbon nanotube-based dry electrode (CG-f@MWCNTs) for electrophysiological signal sensing (reproduced with permission [59] copyright 2019, MDPI AG).

Chemical vapor deposition (CVD) graphene or CNTs grown on Ni or Cu catalyst-based substrates can be patterned using a pattern transferring process, which is more cost- and time-effective than conventional photolithography for the realization of wearable sensor devices [30,38,39,40]. As shown in Figure 2a, a stretchable and transparent graphene-based sensor was directly fabricated on tattoo paper by a simple transfer process, indicating that the target material can be transferred to various substrates using this process [30]. However, there still remains the challenge of causing residual polymers in the pattern transferring process, which is attributed to the use of adhesive polymers such as poly (methyl methacrylate) (PMMA) and thermal release tapes to transfer the target material onto a flexible substrate [40]. The residual polymers can deteriorate the electrical and mechanical properties of the target material. Consequently, with using the pattern-transferring process, additional treatments such as high-temperature thermal annealing and ozone/hydrogen treatment are required to remove the residue, which might lead to the constraint for a wide range of applications [41,42].

#### 2.1.2. Spray Coating and Layer-by-Layer Assembly

The spray-coating process is one of the most commonly used methods for large area deposition using an airbrush, which is an easy, scalable, and low-cost approach for the deposition of the carbon-based films [20,43]. However, the manufacturing accuracy for delivering the carbon material to the desired position is very low due to the use of the airbrush, which increases the processing cost and decreases reliability. To address the issue, Wang et al. used spray deposition modeling (SDM) with a digital x–y plotter combined with a heated substrate, as shown in Figure 2b, to demonstrate CNT-based sensors [44]. Compared to the conventional spray coating method, SDM was able to effectively produce CNTs patterns as well as induce layer-by-layer (LBL) characteristics. Despite the various advantages, there is a limit to the adhesion stability of the deposited film because the adhesion to the substrate is still weak. This implies that the carbon-based film deposited by the spray-coating method requires a specific functionalized substrate for good adhesion. In addition to the simple spray coating method, LBL assembly has been reported as an effective preparation method of manufacturing carbon-based thin-films. This process involves repeated immersion and evaporation and provides alternative deposition of complementary functionalized species on the substrate via various reactions such as electrostatic interactions, hydrogen bonding, or covalent bonding interactions [45]. However, in order to effectively form an LBL carbon-based film with a controllable thickness, the carbon-based materials need to be properly functionalized due to the difficulty of direct assembly with other materials in polar solvents [46]. In addition, it should be taken into account that the methods for the functionalization of carbon-based materials may degrade the electrical properties of the functionalized film by destroying their conjugated structure. Therefore, the considerations for the functionalization of carbon-based materials discussed above are needed to effectively produce a carbon-based film using an LBS assembly.

#### 2.1.3. Screen and Inkjet Printing Process

Printing processes including screen printing, gravure printing, and ink-jet printing have been widely utilized to demonstrate carbon-based electronics and have still drawn tremendous interest for flexible and stretchable carbon-based devices. The main advantage of printing processes is that it can be performed at ambient temperature and pressure, making it an inexpensive and easy manufacturing process [47,48]. Among these printing processes, screen printing is well known as a stencil printing process in which an emulsion screen is used as a template to produce a pattern designed on various substrates, as shown in Figure 2c. This process provides mass production of highly reproducible, disposable, and single-use screen-printed electrodes (SPEs) with low cost [49]. However, since it is hard to produce a desirable carbon-based ink with a controllable viscosity, the printing resolution of screen printing is relatively low. Thus, advanced strategies for carbon-based ink design and printing processes are required to achieve high-resolution printing and fine patterning [50]. For instance, Hyun et al. showed high-resolution patterning of high conductive graphene using a screen printing process with a thinned silicon-based stencil and tuned viscosity of the graphene ink [31].

Inkjet printing enables high-performance wearable carbon-based electronics because small droplets of ink are precisely deposited on the substrate surface. It has several advantages to be used in wearable applications such as high resolution, high throughput, low cost, low processing temperature, and low supplied material consumption [48]. Despite these advantages, jetting performance and compatibility with different depositing inks are still challenges to demonstrating wearable electronic devices using inkjet printing methods. In order to address the issues as well as to manufacture an ideal inkjet-printed carbon-based material in wearable devices, Li et al. demonstrated a stable inkjet-printed graphene with high concentration through a combination of solvent exchange and polymer stabilization [51]. In addition, Hong et al. developed a stretchable CNT-poly(dimethylsiloxane) (PDMS) electrode using both inkjet printing and viscous PDMS controlled by Joule heating on a vertically grown CNT network, as shown in Figure 2d [52].

#### 2.1.4. Drop-Casting and Vacuum Filtration Processes

Solutions made of carbon-based materials can be easily coated and patterned onto substrates by simple process methods such as drop-casting and vacuum filtration without any additional processing [53,54,55]. Drop-casting is one of the simple and low-cost approaches for effectively forming a small-area thin film and is more controllable due to the high dependence of droplets used during the drop-casting process compared to other coating methods [52,56]. However, not only does the approach have limitations of large area coverage, but the uniformity of the films on the substrate is also very low due to the coffee-ring effect [57]. To improve the uniformity of the film deposited by drop-casting, Zhao et al. investigated the interaction between the reduced graphene oxide (rGO) and the substrate surface and showed that the catechol unit effectively affects the uniform rGO film on the PET substrate (Figure 2e), which means that some functionalization of the carbon material or substrate is required to effectively utilize drop-casting with a carbon-based solution [58].

In addition, with the vacuum filtration process, carbon-based film having well-controlled configurational factors such as thickness, width, and length can contribute to manufacturing carbon-based wearable devices on paper substrates. Hossain et al. reported a paper-based flexible dry-electrode for detecting electrocardiogram (ECG) signals by using a vacuum filtration method and a hybrid composite material composed of chemically modified graphene and carboxylic groups-functionalized short multi-walled carbon nanotubes (CG-f@MWCNTs), as shown in Figure 2f [59]. However, this process is limited to those used for filter-paper substrates. In addition, it is still difficult to transfer a thin film coated on paper to a desired substrate or surface that is commonly used in wearable sensors [60].

### 2.2. Carbon-Based Sensors for Wearable Applications

Carbon-based materials are suitable for use in wearable applications due to various advantages such as excellent electrical conductivity, lightweight, high chemical and thermal stability, intrinsic and structural flexibility, ease of chemical functionalization, and potential mass production. Due to these merits, many research groups are consistently developing various sensor devices with high performances, wearability, and reliability using carbon-based materials for practical applications. In this section, we will focus on the design, fabrication, and performance of various types of graphene and CNT-based flexible/stretchable devices, including activity sensors (strain and pressure sensors) and electrophysiological sensors (ECG, EEG, and EMG).

#### 2.2.1. Graphene-Based Materials for Activity Sensors

**Figure 3:**
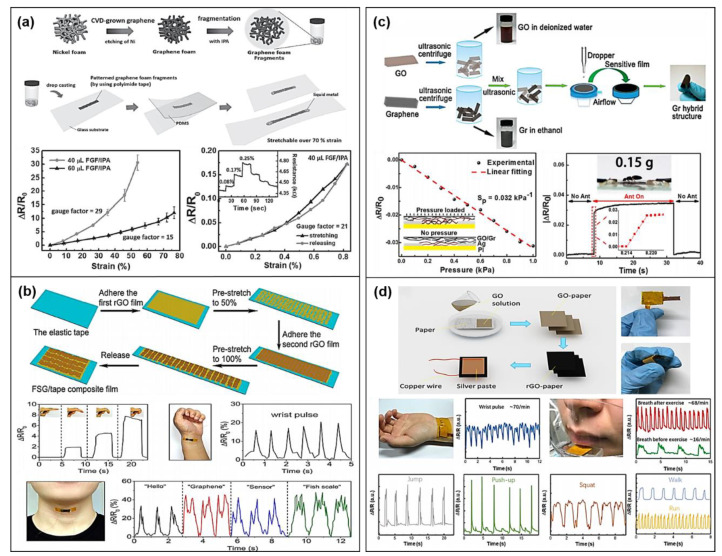
Graphene-based activity sensors. Strain sensors; (**a**) fragmentized graphene foam (FGF) and the relative resistance change of the sensor with different content of FGF with isopropyl alcohol (IPA) under strain (Reproduced with permission [53] copyright 2015, Wiley-VCH). (**b**) Schematic illustration of fabricating a fish-scale-like rGO strain sensor (FSG) and relative resistance responses of FSG strain sensor (reproduced with permission [66] copyright 2016, American Chemical Society). Pressure sensors; (**c**) a hybrid structure GO/Gr (graphene) pressure sensor using graphene oxide-assisted dispersion of graphene and resistive pressure sensor characteristics under pressure (reproduced with permission [73] copyright 2017, American Chemical Society). (**d**) Paper-based flexible pressure sensors for monitoring human activity and their applications for respiration detection, pulse detection, movement monitoring, and voice recognition (reproduced with permission [74] copyright 2017, American Chemical Society).

Wearable activity sensors such as strain and pressure sensors that can be used to measure strain or pressure stimuli by detecting deformation-/pressure-induced electrical signals have attracted attention due to potential applications that can monitor an individual’s movements. In particular, graphene-based materials are widely used in strain and pressure sensors due to their excellent flexibility and good electrical conductivity. However, the pristine graphene produced from the CVD method has low stretchability with a strain of less than 2% [43,61]. Therefore, it is difficult to demonstrate the practical strain sensor using CVD-grown graphene, which suggests that a specific designed structure or configuration for deformable graphene is required for wearable applications [62]. Chen et al. demonstrated a 3D macroscopic structure graphene foam (GF) with a foam-like network using template-directed CVD and showed the improved mechanical and electrical properties of GF [63]. For highly stretchable and sensitive CVD-grown graphene strain sensors, Jeong et al. developed fragmentized graphene foam (FGF) with a large contact area compared to a pristine GF, resulting in high sensitivity with a gauge factor of 29, as shown in Figure 3a [53]. In addition, some research groups demonstrated a highly stretchable strain sensor using buckled graphene on the PDMS substrate and entangled graphene mesh networks, which shows the fully reversible structural deformations (under >30% of strain) and high stretchability (70%) with sensitivity of 2.55 [64,65]. Although highly stretchable graphene was fabricated using a modification of the graphene structure, the process for strain sensors fabricated by the above structural graphene was still expensive and complicated, making it unsuitable for practical applications.

Regarding these issues, graphene materials obtained from graphite oxide are promising materials for strain sensors due to their easy fabrication with different structures and facile accessibility with other materials. Liu et al. reported a high-performance strain sensor with a fish-scale-like reduced graphene oxide (rGO) film on an elastic tape using stretching/releasing the composite films of rGO/tape bilayer, as shown in Figure 3b [66]. In the study, the fabricated strain sensor exhibited a broad strain sensing range (82%) and is able to monitor finger motion, wrist pulse, and phonations due to high sensitivity. Moreover, high stretchable and sensitive rGO-based strain sensors have also been developed using different forms as sensing materials, such as rGO-/DI (deionized water)-conductive liquids and rGO microtubes–elastomer composites, resulting in detection of physical human motions [67,68]. In addition to the modification of graphene structure or phase, Wu et al. added metal nanowires (AgNWs) into the GF to form a hybrid foam with a 3D ordered microstructure, and then the strain sensor with the hybrid foam exhibited high conductivity and excellent deformability, providing great potential for wearable applications [69]. For graphene-based strain sensors on a fabric substrate, rGO and graphene plates were formed by using a dipping and LBL coating process, and the fabricated strain sensors successfully monitored various activities of human such as abdominal breathing, joint movement, and swallowing [46,70].

For graphene-based pressure sensors, several researchers have developed advanced fabrication technologies and functional materials combined with graphene. Xia et al. demonstrated a CVD graphene pressure sensor on PDMS substrates using a directly CVD-grown approach on a highly deformable substrate [71]. The sensor exhibited a pressure range of 0.2–75 kPa with a high sensitivity of 110 kPa^−1^, causing CVD graphene-based pressure sensors to show a good performance in detecting the human activities [72]. However, the CVD approach not only is well known for its expensive processes but also requires complex processes to form and pattern graphene on stretchable and flexible substrates. Thus, to address the issues, a solution-processed hybrid structure composite was developed using both graphene oxide-assisted dispersion and graphene, and the fabricated pressure sensor exhibited a sensitivity of 0.032 kPa^−1^ in the low pressure range up to 1 kPa, as shown in Figure 3c [73]. In addition, as shown in Figure 3d, Tao et al. proposed a paper-based flexible pressure sensor for monitoring human activities such as respiration detection, pulse detection, movement monitoring, and voice recognition using rGO solutions and inexpensive paper, and the fabricated pressure sensor showed significant performance improvements in the pressure range (20 kPa) and sensitivity (17.2 kPa^−1^) [74], which indicates that a highly performance graphene-based pressure sensor can be successfully demonstrated using low-cost approaches.

#### 2.2.2. CNTs-Based Materials for Activity Sensors

**Figure 4:**
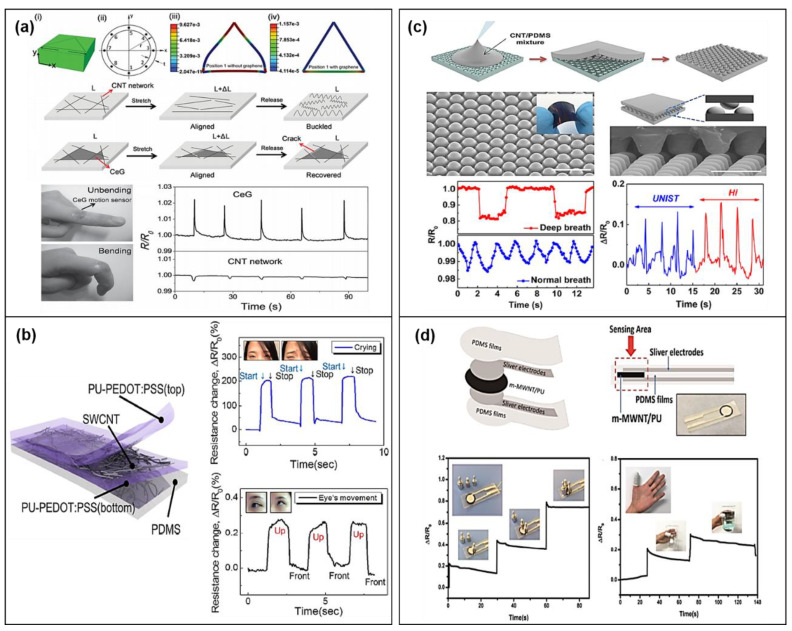
Illustration and characterizations of CNT-based strain and pressure sensors. (**a**) Strain sensors with graphene reinforced in the CNT network and stretchable wearable applications in finger motion detection (reproduced with permission [79] copyright 2016, Wiley-VCH). (**b**) Structure of the strain sensor consisting of the three-layer stacked nanohybrid structure of PU-PEDOT:PSS/single-walled carbon nanotube (SWCNT)/PU-PEDOT:PSS on a PDMS substrate and the responses of the sensor for subtle changes such as emotional expressions and eye movements (reproduced with permission [34] copyright 2015, American Chemical Society). (**c**) Schematic showing fabrication and structure of a microdome-shaped CNT-PDMS composite/sensor and the applications in detection of human breathing flows and voice vibrations (reproduced with permission [84] copyright 2014, American Chemical Society). (**d**) Photograph of the highly stable and flexible pressure sensor and relative change of resistance for static loading pressures and subject’s finger gestures (reproduced with permission [35] copyright 2018, MDPI AG).

The structurally modified form of CNTs is a common approach for making highly stretchable and flexible wearable activity sensors. For CNTs-based sensors with high electrical and mechanical properties, advanced technologies have been developed by various research groups. Yamada et al. developed a vertically aligned single-walled carbon nanotubes (SWCNTs) film on stretchable substrates using a CVD process for high resistance against strain up to 280% and showed applicability in human motion detection [75]. In addition, ultrahigh stretchable and wearable devices were demonstrated using dry-spun CNT fibers directly on pre-strained eco-flex substrates, and the fabricated strain sensor can be stretched by over 900%, providing high sensitivity of 0.56 and 47 for strains of 200% and 440%, respectively [76]. However, although the CNTs-based strain sensors capable of forming 1-dimensional (1D) conductive networks are favorable to be highly stretchable strain sensors for detecting large strain ranges, such sensors still exhibited low reliability and poor stability under stretching/releasing cyclic test, owing to easy current breakdown and low reproduction quality [77]. For cost-effective and durable CNTs-based strain sensors, Ahn et al. proposed a simple and scalable template-guided self-assembly for the integration of SWCNTs into non-random 2D rhombic nanomesh, which improves their durability as well as stretchability [78]. Additionally, as shown in Figure 4a, to improve the mechanical strength and electrical conductivity of the CNTs material, graphene was reinforced in the CNTs, where the graphene homogeneously fills the nanotube voids and forms a seamless hybrid, and the fabricated strain sensor by graphene-reinforced CNT networks showed high resistance to buckling or bundling stress in large cyclic strain tests (up to 20%) [79]. In addition to high mechanical characteristics, the linearity and detection ability of the strain sensor for small stimuli are important parameters for accurately detecting various changes in strain and expanding the applications. Zhang et al. reported a nano-composite material (Ag@CNTs) composed of silver nanoparticles (AgNPs) and CNTs for high stretchability and good linearity of the strain sensor [80]. Moreover, as shown in Figure 4b, Roh et al. demonstrated a transparent, stretchable, ultrasensitive, and tunable strain sensors using a sandwich-like stacked monohydride film including SWCNT and a conductive elastomeric composite with polyurethane (PU) and poly(3,4-ethylenedioxythiophene) polystyrenesulfonate (PEDOT:PSS) for detection of small strains, and the fabricated sensor exhibited very high sensitivity of 837.1 under 3.5% of strain, allowing it to detect subtle changes such as emotional expression and eye movement in different directions [34].

Besides the flexible/stretchable CNTs-based strain sensors, CNTs have been widely used in wearable pressure sensors. Wang et al. demonstrated SWCNT’s pressure sensor with the sensitivity of 1.8 kPa^−1^ within 300 Pa using a silk-molded micro-patterned PMDS substrate, and the pressure sensor showed the capability of monitoring small signals in humans such as speech recognition and wrist pulse detection [81]. Zhan et al. also reported a pressure sensor based on SWCTN/tissue paper. Zhan et al. assembled the SWCNT/tissue paper on the Au/polyimide (PI) substrate and sealed the device using a PDMS layer to provide mechanical strength [82]. The pressure sensor showed a wide range of detection (2.5–35 kPa) with a sensitivity of 2.2 kPa^−1^ and the potential to monitor human physiological signals.

In general, CNTs can provide high-pressure sensitivity, but the interface between the flexible substrates and the CNT materials is not strong enough without specific treatments, resulting in low stability of the pressure sensors. To address this issue, a combination of 1D CNTs and 2-dimensional (2D) graphene materials can be utilized as an alternative method, because it can cause a large contact area with the substrate. Jian et al. demonstrated a CNT/graphene hybrid film aligned (ACNT/Gr) on a micro-structured PDMS (m-PDMS) film molded directly from a natural leaf for a high-performance wearable pressure sensor. The ACNT/Gr hybrid composite film not only helped the pressure sensor to detect bending, torsion, and acoustic signals but also helped to have a high sensitivity of 19.8 kPa^−1^ (<0.3 kPa) and high stability (>35,000 cycles) [83]. In addition, as shown in Figure 4c,d, by adjusting the dispersion/neighboring interaction between the CNTs and the polymer matrix, the functionality of the CNTs-polymer composite was improved to demonstrate a highly stable and flexible/stretchable pressure sensor [35,84]. In this regard, a micro dome-shaped CNT-PDMS composite film was used for the detection of human respiratory flows and voice vibration. The fabricated pressure sensor showed a minimum detection limit of 0.2 Pa and a sensitivity of 15.2 kPa^−1^ with a fast response/recovery time (~0.04 s) [84]. He et al. also used a modified multi-walled carbon nanotube (m-MWCNT)/PU composite and a PDMS’s encapsulation layer, and the fabricated pressure sensor exhibited a sensitivity of 4.282 kPa^−1^ within 1 kPa and successfully detected finger gestures, respiratory, and pulse of the subject [35].

#### 2.2.3. Carbon-Based Materials for Electrophysiological Sensors

**Figure 5:**
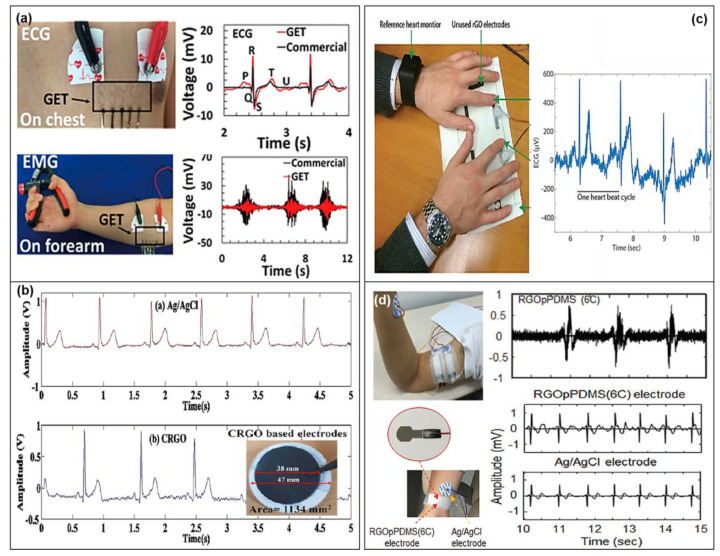
Graphene-based electrophysiological sensors. (**a**) The measurement of ECG and EMG signals by graphene electronic tattoo (GET) sensors attached on chest and forearm, respectively (reproduced with permission [30] copyright 2017, American Chemical Society). (**b**) ECG measurement using a commercial electrode (Ag/AgCl) and chemically reduced graphene oxide-based (CRGO) dry electrodes on paper substrates (reproduced with permission [92] copyright 2017, Elsevier). (**c**) Measuring ECG signal with two fingers placed on printed graphene electrode on fabric substrates (reproduced with permission [93] copyright 2017, Royal Society of Chemistry). (**d**) EMG signals recorded from biceps brachii muscle and ECG signals recorded from right arm using rGOpPDMS bioelectrodes (reproduced with permission [94] copyright 2017, Wiley-VCH).

It is well known that graphene is a 2D material possessing good physical, electrical, chemical, and mechanical properties such as high conductivity, high mechanical strength, excellent flexibility, and ease of functionalization. Among various advantages, the remarkable parameters of graphene are high electrical and mechanical properties including very high electron mobility of ~200,000 cm^2^/V·s, the thermal conductivity of 5300 W.m^−1^.K^−1^, surface area to volume ratio of 2630 m^2^/g, Young’s modulus of 0.5 TPa, and resistivity of 10^−6^ Ω.cm [85,86,87,88]. Due to these excellent properties, graphene is often considered as a strong candidate for wearable health care electrophysiological sensors. Recently, Ameri et al. designed and fabricated a CVD grown graphene-based tattoo sensor for ECG measurement. As shown in Figure 5a, the graphene electronic tattoo (GET) sensor was able to measure or detect the ECG signal from the subject’s chest, and the ECG signal level extracted by the graphene-based tattoo sensor is much higher than that of the commercial ECG sensor. In addition to ECG detection, EMG with low signal-to-noise ratio (SNR), electroencephalogram (EEG), skin temperature, and skin hydration are available to be detected by GET sensor [30]. Similarly, Lou et al. [89] and Celik et al. [90] demonstrated ECG sensors on flexible and rigid substrates using CVD grown graphene, respectively. The quality of the ECG signals is clearer and less noisy than a commercial gel-type electrode. However, these CVD grown processes for demonstrating graphene-based electrophysiological sensors in practical biomedical applications are still challenging because of their high manufacturing cost for large areas and the problems with microscale-patterning and direct transfer to skin-like soft substrates. With the intention of overcoming the aforementioned limitations, various approaches have been developed for manufacturing modified graphene-based materials such as rGO and chemically modified graphene (CG) via chemical reduction, thermal reduction, electrochemical reduction, photo-catalyst reduction, and hydrothermal/solvothermal reduction [88,91]. In addition, these graphene materials are considered as a promising alternative for a wide range of applications due to their flexibility, scalability, and adaptability.

For high biocompatibility and good biopotential signal, Hossain et al. [88] and Das et al. [92] proposed a paper-based ECG sensor with good skin-electrode contact. As shown in Figure 5b, they fabricated ECG sensors on a nylon filter paper using rGO suspension and vacuum filtration, and the ECG sensor with circular-shaped dry electrodes obtained high-quality ECG signals from the fingertips [88,92]. This process is very easy and convenient for large-scale production of graphene-based ECG sensors. Furthermore, rGO can be formed on various types of flexible/stretchable substrates such as fibers, fabrics, and polymers substrates and is easy to be functionalized with other carbon materials and metal nanoparticles. Due to these advantages, these rGO materials are widely used in wearable physiological sensors. As shown in Figure 5c, for demonstration of a fabric-based ECG sensor, an rGO film was formed and patterned on a fabric substrate using an inkjet printer, and the prepared dry rGO electrode was able to detect ECG signals from the fingertips [93]. Moreover, in order to monitor diverse bio-signals like ECG, EMG, and EEG without wet-gel, Yun et al. demonstrated a high performance epidermal electronic device consisting of rGO sheet on a porous PDMS thin-film using a simple solution-processed method. In the study, the ECG signal was successfully monitored on the wrist as well as the EMG signal from the upper arm, as shown in Figure 5d, indicating that the signal quality and amplitude are quite similar to the commercial wet electrodes [94].

CNTs material is also a promising candidate for wearable physiological sensors because of their large aspect ratio, great flexibility, extremely high tensile modulus and strength, and excellent electronic properties [95,96,97]. Kang et al. proposed a CNT-based wearable physiological sensor using a PDMS mold with a metal snap connector and CNT paste. To improve the conductivity of a flexible CNT-based dry electrode for physiological signal detection, concentration optimization in a CNT dispersion solution with polymer matrix was implemented, and then the optimized dry electrode could measure both ECG and EMG signals without conductive gel. The study showed that the signal intensity recorded from the CNT-based dry electrode was comparable to that of the commercial Ag/AgCl wet electrodes. In addition, the study measured the EMG signal coming from the forearm, and the SNR of the CNTs-based EMG sensor was superior to that of commercial electrodes [97].

However, in the case of CNT-based polymer composites for wearable physiological sensors, CNT’s agglomeration in the viscoelastic polymer matrix is still a challenging issue, which can severely reduce electrical characteristics. To avoid the issue, several endeavors have been performed to promote the dispersion of CNT particles into the polymer, including surface modification, shear mixing, mechanical agitation, ultrasonic treatment, and ball- or micro-bead milling [98,99,100]. Kim et al. reported methyl group-terminated PDMS with CNTs and obtained good electrical properties of the composite. Using highly conductive and elastic composites developed in a simple and cost-effective way, ECG and EMG measurements can be performed, and then high-quality signals were achieved [101].

It is also worth considering long-term usability and biocompatibility with human skin to use CNT-based materials for physiological signal monitoring. Jung et al. investigated the effect of composition (concentration of CTNs) and the size of the CNT/PDMS electrode on signal quality for long-term ECG monitoring. The optimized composite electrode (4.5 wt%) showed the ECGs with good quality and no degradation over time compared to Ag/AgCl electrodes due to its good biocompatibility and robustness under an extreme environment including movement and sweat [102]. Additionally, in order to achieve a high quality of bioelectrical signals, wearable physiological sensors need to be conformally laminated onto the skin. Lee et al. demonstrated self-adhesive CNT-based electronics with adhesive PDMS materials, and the electronics exhibited long-term continuous recording of ECG signals without skin irritation due to their great conformal contact and biocompatibility [103].

## 3. Textile-Based Wearable Sensors

Textiles are spotlighted as candidates for wearable sensors capable of detecting various electrophysiological signals and human activity due to their mechanical properties such as flexibility and stretchability, wearability, breathability, and user comfort. The approach to embedding sensing elements into textiles allows long-term monitoring without discomfort and the freedom of sensor configuration depending on the different usage. In addition, textile-based sensors are compatible with traditional textile manufacturing processes, resulting in scalable and cost-effective producing of sensors [104,105,106].

This section describes mainly functional materials and fabrication technologies for demonstrating textile-based sensor devices and their applications. Electrical/mechanical properties and the remaining challenges for textile-based wearable sensors were also discussed. Furthermore, it shows effective strategies to realize textile-based sensors and the possibility of next-generation textile-based wearable sensor systems by reviewing overall research for various textile-based electronics.

### 3.1. Fabrication Technology

#### 3.1.1. A Simple Coating Processes; Dipping and Drying

**Figure 6:**
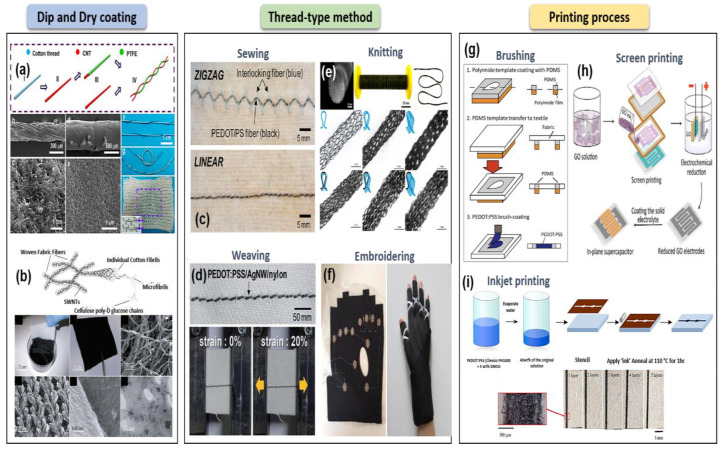
Fabrication technologies for textile-based wearable sensors. Dip and dry coating; (**a**) fabrication of fiber-based electronics with CNT-coated cotton thread (CCT) using a dipping and drying process (reproduced with permission [107] copyright 2014, American Chemical Society). (**b**) The SWCNT-coated woven polyester fabric using an extremely simple dipping and drying process (reproduced with permission [32] copyright 2010, American Chemical Society). (**c**) Optical images of zigzag- and linear-type PEDOT/PS fiber embedded fabrics by sewing method (reproduced with permission [33] copyright 2017, American Chemical Society). (**d**) Woven sensor fabric fabricated by weaving process (reproduced with permission [118] copyright 2017, Royal Society of Chemistry). (**e**) PU/PEDOT:PSS fibers co-knitted with a commercial Spandex yarn using knitting method (reproduced with permission [121] copyright 2015, American Chemical Society). (**f**) Textile-based smart gloves realized by the embroidering method (reproduced with permission [122] copyright 2017, Informa UK Limited). Printing process; (**g**) schematics of the fabrication process of brush-painted PEDOD:PSS electrode on knitted fabrics using PDMS-made stencil (reproduced with permission [125] copyright 2015, Nature Publishing Group). (**h**) Screen-printed rGO electrode on woven cotton fabrics through the strong chemical interaction between GO and cotton fibers (reproduced with permission [126] copyright 2017, IOP Publishing). (**i**) Patterning PEDOT:PSS on PET nonwoven fabric using inkjet printing and sponge stencil (reproduced with permission [128] copyright 2016, American Chemical Society).

The “dip and dry” coating method is a very simple process to deposit the desired films on fabrics (Figure 6a,b) [32,107]. Unlike vacuum-based deposition processes such as CVD, atomic layer deposition (ALD), and sputtering processes, this approach allows a solution-based process that could provide low-cost, large-area application, and high throughput. Despite various advantages, there are still some challenges such as weak adhesion between the deposited film and fabric, complicated step to get appropriate electrical and mechanical properties, and patterning [108,109].

Using a simple and continuous dipping and pad-dry process, a stable reduced graphene oxide (rGO)-coated cotton textile fabric has been demonstrated [110]. Compared to the traditional process of textile-coating, which is complex and difficult for larger area application, the proposed process is scalable and cost-effective for producing rGO-coated fabrics. In addition, the rGO textiles exhibited good mechanical properties such as flexibility and tensile strength and durability for washing. However, although the study improved the interface adhesion through interaction with the functional group of fabrics and the negative charge of GO, it still not enough to achieve strong adhesion between the fabric surface and the deposited materials, resulting in poor electrical characteristics [110]. To address this issue, highly conductive single-walled carbon nanotubes (SWCNTs)-woven polyester fabrics having a strong bond with the fabric were fabricated by introducing additional treatments such as acid and thermal reaction [32]. In addition to good adhesion between the conductive material and the fabric substrate, high mechanical properties and conductivity are also very important parameters for textile-based wearable applications. For the fabrics or fibers with high conductivity and flexibility/stretchability, all-carbon composite fabrics made of activated carbon fiber felt (ACFF) body material and nanoscale CNT or graphene (as a conductive filler) were developed using a simple impregnation and freeze-dying method. Moreover, various materials such as PEDOT-PSS, graphene, and rGO for a simple soaking or dipping process were used to improve the electrical and mechanical characteristics of textile-based materials [111,112,113,114,115]. However, patterning a conductive textile-based material is still a challenging task for practical wearable sensors.

#### 3.1.2. Thread-Type Processes; Knitting, Weaving, and Embroidering

For miniaturization of wearable textile-based sensors, thread-type methods such as knitting, weaving, and embroidering are continuously developed (Figure 6c–f). These coating technologies are solvent-free and compatible with the conventional textile manufacturing equipment, resulting in small dimensions, various desired shape patterns, and good wearability. Furthermore, according to topology or structure of threads connected with insulating fibers or fabrics, their electrical and mechanical properties can be controllable. Therefore, it is easy to integrate them with various electronic systems. However, thread-type sensors are tough to withstand certain mechanical deformations and to maintain their electrical characteristics during textile processing, which means that the selection of materials used is limited.

In order to achieve a softer structure and stretchability, Huang et al. demonstrated a yarn-based piezoresistive sensor by wrapping both a composite core yarn composed of polyester fiber and elastic fiber and a piezoresistive fiber (carbon-coated fiber) into a skein [116]. A similar approach is used by Tai et al., where they used double-twisted smart fibers made of SWCNT-coated conductive fibers with homogeneous thickness and graded thicknesses to detect an applied load and to identify the position of the load [117]. Although these approaches are able to demonstrate thread-like stretchable sensors, their corresponding electrical performances of conductive fibers are still low, causing low sensitivity and a limitation for a wide range of applications. One of the merits of the thread-type processes is that sensor devices and fabrics can be easily integrated by conventional textile manufacturing methods. For thread-type wearable strain sensors capable of detecting various human motions, the graphene-coated conductive yarns were embedded in an elastomeric medical patch or textile products using an easy and simple weaving process [46]. In addition, Eom et al. reported a polymerized PEDOT/Polyester (PS) fiber-based body-motion sensor with multi-functionality using a sewing method. This study especially showed that a fiber pattern design by the sewing process can control sensitivity and sensing range of the sensors [33]. Thus, the weaving or sewing process appears to be easy-access to textile-based sensor applications with easy and simple integration with fabrics and yarns [118,119]. Similar to the fiber pattern design, Maziz et al. reported that textile architectures with woven and knitted structures can control or improve output force and strain, which indicates that the electrical performances of textile-based devices can be optimized by applying different textile architectures [120]. Moreover, using co-knitting with a conductive polymer fiber and a commercial spandex yarn, the textile sensor’s performances such as stability and durability were improved [121]. However, the knitted fabrics are highly dependent on the structure of the knitted fabric, causing the application to other types of fabrics to be rather limited.

In addition to weaving and knitting technologies, the embroidering method has been used to demonstrate textile-based wearable sensors [122,123]. In particular, textile-based radio-frequency (RF) applications made of silver-coated conductive fibers on the polyester fabric was demonstrated using the embroidering process. In this work, to enhance various critical elements such as the electrical conductivity, mechanical properties, inherent chemical stability, and water resistance for wearable applications, both the double-layer embroidery and PDMS substrate were applied. However, this approach is not suitable for large-area applications because of its great need for materials and their (embroidered fibers’) stiff mechanical property [123].

#### 3.1.3. Printing Process; Stamp-Transfer, Stencil, and Screen Printing

The printing process is a promising technology for patterning conductive materials on fabrics without the need for photolithography and chemical etching. Compared to other fabrication technologies, this printing approach is more cost-effective and high-throughput and can be printed on a wide variety of substrates with the desired various shape patterns (Figure 6g–i). However, viscous conductive composite was substantially required to print it on fabrics directly. In addition, in order to avoid delamination or crack of the printed patterns against mechanical deformations, the degree of penetration to the fabrics for viscous composite should be carefully considered. Otherwise, a nonconductive encapsulation layer is necessary to protect materials printed on a fabric substrate.

With considering the permeation of viscous conductive ink on fabrics, Jin et al. coated textile-permeable conductive ink directly on textile substrates using a screen/stencil printing process. The mechanical and electrical properties were controlled by the evaporation rate of ink’s solvents. As a result, the printed textiles exhibited excellent durability and stretchability in the demonstration of a four-channel wireless EMG monitoring garment [124]. Similarly, a simple and reliable printing process for PEDOT:PSS reported by Takamatsu et al. has been used to create a desirable pattern on fabrics. The PEDOT:PSS used in the study was brush-coated and patterned on a knitted fabric using a PDMS-made stencil, and the fabricated PEDOT:PSS-based fabric sensor with an ionic liquid gel exhibited high-quality ECG recording signals even during movements [125]. In contrast to using an optimized viscous conductive ink, rGO was screen-printed on a woven cotton fabric through the strong chemical interaction between GO and cotton fibers. As a result, the printed rGO electrode showed good mechanical properties [126]. In order to improve uniformity and adhesion between printed electrodes and fabrics, an interface layer was adopted on the surface of fabrics before printing conductive materials. The printed electrodes on fabrics with interface layer can lead to high electrical as well as mechanical properties due to their smooth and flexible surface layer, resulting in good applicability in various textile substrates with different structures [127].

Inkjet printing can also be used for forming and patterning conductive materials on fabrics, and the viscosity of conductive ink for inkjet printing should also be optimized for printing on fabrics. Guo et al. reported all-organic conductive wires patterned on the PET fabric using a control of concentration of PEDOT:PSS and inkjet printing with a sponge stencil [128]. However, due to its incompatibility with non-planar and large-sized substrates, there is a limit to the wide range of usability for inkjet printing. Thus, for addressing challenges of the printing process, a facile stamp transfer method is considered as an alternative due to the feasibility of printable sensors without a limitation for substrate surfaces being used [129].

### 3.2. Sensor Devices for Healthcare Monitoring

For the feasibility of the textile-based wearable sensors, several research groups have developed various sensor devices on textile-based substrates such as strain, pressure, and electrophysiological sensing devices, which have mainly been demonstrated by two different architectures. One is embedding textile-based sensors on a premade or weaved planar fabric [130,131,132]. The other is demonstrating thread-like (cylindrical) functional sensors on a single fiber [133,134,135]. However, although textile-based sensor devices in one- or two-dimensional configuration can lead to various applications without platform limitations, their electrical performances are still unsatisfactory for practical applications of consumer-level sensor systems, compared to that made on common planar substrates such as silicon and flexible plastic films. Therefore, new designs for structures and materials and effective technologies for the fabrication process and integration with other electronics should be developed and considered as important factors for the realization of textile-based wearable sensors.

#### 3.2.1. Activity Sensors; Strain and Pressure

Textile-based wearable activity sensors such as strain and pressure sensors are being recently developed for monitoring various health-related biometric parameters due to their wearable comfort and unique properties. In general, the sensing mechanism of activity sensors is that mechanical deformations are converted into electrical signals. Accordingly, the sensor with high sensitivity and mechanical properties is required to detect changes in a wide range of deformations, which can lead to the precise measurement of the health status of humans. Various textile-types of strain and pressure sensors have been demonstrated as listed in Table 1.

For textile-based pressure sensors, various conductive materials such as rGO [136], CB/PDMS composite [137], and PEDOT:PSS/PVDF [138] and structures such as 2D-stacked [36], 3D spacer [139], and cross-contact [140] were used to detect the applied pressures. Liu et al. reported a flexible rGO pressure sensor with multi-layer stacked structures on a silk fabric via the thermal reduction process. The fabricated sensor can detect different pressures and subtle body pressure as well. To improve the mechanical properties of the sensor, the rGO material was encapsulated using the PI tape as a protective layer, but it may lose the inherent fabric properties such as wearability and stretchability [136]. In addition, by using three-dimensional (3D) spacer fabrics as a unique textile sensing structure, a novel warp-knitted 3D spacer fabric pressure sensors with a sensitivity of 50.3 × 10^−3^ kPa^−1^ and good reliability/stability were successfully demonstrated [139].

**Figure 7:**
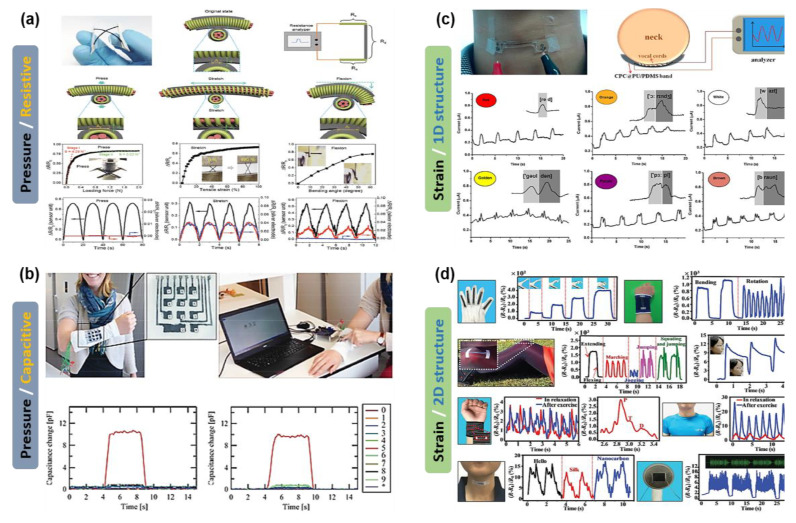
Textile-based wearable sensors for pressure and strain. (**a**) Highly stretchable sensor units/arrays with the intertwined composite fibers and piezoresistive rubber and mechanical–electric characteristics (reproduced with permission [137] copyright 2016, Wiley-VCH). (**b**) The stretchable keyboard on a knitted fabric using capacitance-type pressure sensor made of PDMS film and printed PEDOT:PSS electrodes and the changes in capacitance value against pressure (reproduced with permission [36] copyright 2016, Wiley-VCH). (**c**) Conductive polymer composites made of carbon black on a polyurethane (CPC@PU yarn) strain sensors attached on the neck and the current changes as the function of the pronouncing different words (reproduced with permission [141] copyright 2016 American Chemical Society). (**d**) Ultra-stretchable carbonized silk fiber-based strain sensors and various applications in human motions and sounds (reproduced with permission [143] copyright 2016, Wiley-VCH).

In addition to the demonstration of a sensor unit, a sensor array is required to obtain high-resolution detection characteristics and spatial distribution mapping of various mechanical stimuli. As shown in Figure 7a, the intertwined composite fibers and piezoresistive rubber made of PDMS and CB were used to demonstrate a highly stretchable sensor array. The sensor array not only can simultaneously map and quantify the mechanical deformations such as pressure, lateral strain, and flexion but also can detect the detailed diagnostic information of human pulse wave. Compared to the previously reported pressure sensor, it is able to distinguish between the different types of mechanical deformations, mainly owing to their cross-contact configuration [137]. In addition, Figure 7b shows a stretchable keyboard on a knitted fabric with a capacitance-type pressure sensor array. In this work, a PDMS film and PEDOT:PSS electrodes printed by a PDMS stencil and the brushing process were used to fabricate the textile-based keyboard capable of reading pressure applied from a human finger, and the sensor also exhibited good mechanical and electrical characteristics [36].

For a textile-based strain sensor, the elastic properties are very important to realize stable and durable sensor devices under the deformation. Most of the reported textile-based strain sensors used elastomer-based composites and fibers as sensing elements and substrates, respectively to guarantee high stretchability. Wu et al. used a conductive polymer composite (CPC) made of CB and natural rubber on a polyurethane (PU) yarn (CPC@PU) to achieve a high sensitivity. This CPC@PU yarn strain sensor showed good reproducibility and high sensitivity and could detect wrist pulse waves and delicate motion of throat during pronouncing, as shown in Figure 7c [141]. In addition, by webbing both a conductive yarn in the weft and an elastic yarn in the warp, a wearable textile-based gesture sensor was demonstrated, and the sensor could monitor the flexion angles during elbow and knee movements [142]. Furthermore, as shown in Figure 7d, in order to fabricate an ultra-stretchable textile-based strain sensor, a plain-weave carbonized silk fabric was used, resulting in the carbonized silk-based sensor exhibiting high sensitivity (GF = 37.5) and super stretchability (500%). Owing to their high performances, this sensor can detect various human motions such as jogging, bending, jumping, and gaiting, as well as sounds [143]. A wireless wearable musical instrument prototype and a thread-like (1D) strain sensor demonstrated using a combined CVD-graphene with a woven fabric (GWF) and graphite nanoplatelets (GNPs), respectively [144,145]. These sensors enable the mapping of the strain states and showed excellent durability against mechanical deformations.

Although the advances are noteworthy, the capability to distinguish multiple forms of deformation for a textile-based strain sensor is still needed to realize practical applications. Recently, for discrimination of various stimuli such as tensile strain, bending, and torsion, graphene-based fiber sensors were fabricated using a polyester-winded PU core fiber with a compression spring structure. The fiber strain sensor exhibited excellent stretchability (200%) and sensitivity (GF = 35) [146]. In addition, to distinguish hand motions and grasps, Heo et al. reported an approach that utilizes both the PDMS interface and passivation layers on fabric substrates and then demonstrated a textile-based glove sensor on a polyester-based spandex fabric substrate, resulting in the detection of human hand motions and distinguishing 3D-printed prosthetic hand grasps [147].

#### 3.2.2. Biophysiological Sensors; ECG, EMG, EEG, Sweat, and Body Temperature

In order to measure or record biophysiological signals such as ECG, EEG, EMG, sweat, and temperature, the sensor needs to be in comfortable contact with the skin for long-term use. The textile-based structure/configuration is suitable for biophysiological sensors because the textile provides hygroscopic and breathable properties and compatibility with human skin.

**Figure 8:**
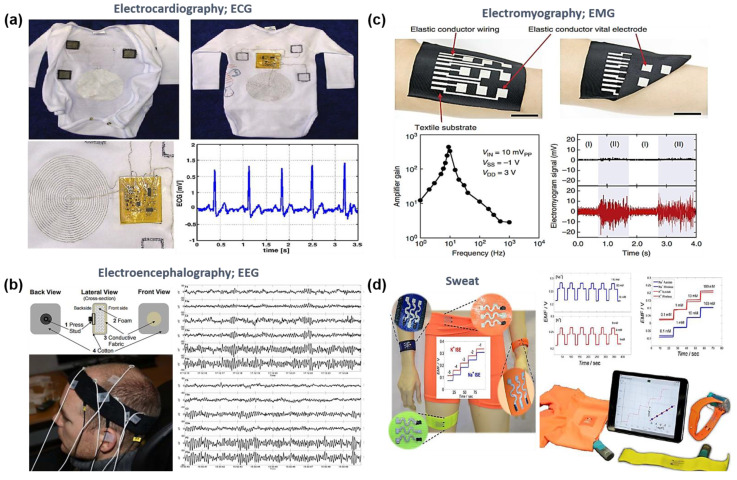
Textile-based wearable sensors for biophysiological signals. (**a**) Textile-based baby suit prototype with the embroidered coil for monitoring a continuous ECG signal (reproduced with permission [149] copyright 2006 Elsevier). (**b**) The hair bandage-type EEG signal sensor using soft and tender textile-based electrodes and EEG signals (reproduced with permission [151] copyright 2012, MDPI AG). (**c**) Measurement of arm EMG signals with an elastic conductor electronic textile and EMG signals obtained through the elastic conductor attached to a forearm while opening (I) and closing a hand (II) (reproduced with permission [153] copyright 2015, Springer Nature). (**d**) A textile-based wearable multi-ion sweat sensor array using composites with PU-based ion-selective membranes and CNT inks for detecting the change in concentration of sodium and potassium (reproduced with permission [37] copyright 2017, Wiley-VCH).

For a demonstration of the ECG sensor (Figure 8a), Tada et al. used silver- and carbon-based composite inks as the connection wires, which were waterproof and with excellent mechanical properties. The inks were directly printed on an undershirt to measure the ECG signals. The ECG signal can be accurately measured in exposing perspiration because a conductive wire was not affected by a high-humidity condition. However, it has low durability of the ink wire against deformations, resulting in a limit of long-term use [148]. To address this issue, a garment-embedded patient-monitoring system was introduced using a knitting technology. The study showed a continuous ECG signal monitoring for a long time with higher SNR [149]. Tsukada et al. also reported that continuous ECG signals from an anesthetized rat were successfully recorded using a PEDOT-PSS glycerol thread without any gel-type interfacial adhesive [150].

Along with long-term use, the comfortability for the user is considered as a key factor for the realization of textile-based health-monitoring electronics. As shown in Figure 8b, a comfortable EEG hair bandage-type sensors were reported in which the EEG sensors can improve the clinical situation, compared to the existing caps made of stiff electrodes causing the skin irritation. However, although the proposed textile-based sensor can continuously record the EEG signals, it required a use of electrolyte gel or saline solution for monitoring good-quality EEG signal [151,152].

For textile-based EMG sensors, stencil printing with highly conductor ink consisting of Ag flakes and fluorine rubber/surfactant is utilized to integrate sensor devices into the fabrics, as shown in Figure 8c [153]. Dias et al. also reported that a printable elastic conductor with high conductivity and a conductive yarn sewn with leggings were used as a textile electrode to demonstrate a wearable sensor [154]. With both highly elastic conductive inks and textile substrates, the textile conductive electrodes successfully captured the EMG signal and subsequently transferred it to the sensor electronics [153,154]. In addition, Linz et al. reported the contactless EMG sensors embroidered onto fabric using conductive yarns to avoid a problem related to the interface potential between the skin and the electrode, and the fabrics embroidered sensors with contactless electrode provided similar EMG signals and shapes compared to the commercial EMG electrodes [155]. However, the electronics integrated into the fabric for data acquisition are still rigid or stiff, leading to difficulty for demonstrating the fully flexible wearable sensor system.

The monitoring of sweat rate and contents can give us valuable information for healthcare. For example, the sweat rate can be used for monitoring dehydration levels and preventing overheating and associated heat-related illnesses. A textile-based wearable multi-ion sweat sensor array was demonstrated using a composite with PU-based ion-selective membranes and CNT inks, and a screen-printing method (Figure 8d). The fabricated sensors not only have high mechanical properties but also the detection ability for the change in concentration of sodium and potassium [37]. The previous sweat sensors have problems such as creating capsules on the skin surface, which cause variation of the sweat composition and complex circuitry. In order to address these issues, a wearable sensing system was proposed for sample collection and transport with various sensors. This study reported a wearable multi-parametric patch-type sensor on a fabric, and the sensor can detect real-time changes in sodium concentration, the conductivity of sweat, and pH during exercise [156].

Textile, in particular with a form of garments, is the most suitable platform to monitor body temperature. Sibinski et al. [157] and Shang et al. [158] demonstrated a thread-like (1D) temperature sensor using a PVDF monofilament fiber and double-helix CNT yarns, respectively. Moreover, using the polymer composite filled with MWCNTs as a thermo-sensing material, a thread-like temperature sensor with high mechanical properties and TCR of 0.13%/K on the PVDF monofilament fiber was demonstrated [157]. In addition, a multifunctional temperature sensor with the structure of double-helix CNT yarns was reported, and the sensor could detect the reversible changes in resistances for strains or stresses, light illumination, and environmental temperature due to hierarchical helical yarns. The study showed the resistance of the sensor also increased with calculated slopes in the range of 0.080.14 Ω/°C as temperature increased. However, shrinkage and slight deformation of the double-helix CNT yarn occurred when temperature decreased by cooling to recover the initial resistance value, which results in the hysteresis of temperature reading [158].

## 4. Challenging Issues and Future Routes; Carbon- and Textile-Based Wearable Sensors

Carbon-based materials show many advantages to be used in the development of various wearable sensors, but there are still challenges in terms of compact design, low-cost fabrications, device protection layers, multi-functional sensing, and integration techniques for realizing fully embedded carbon-based practical wearable electronics. In particular, low-cost and mass production of carbon-based materials with high performances are most demanding. Although various effective processes have been studied for cost-effective and mass preparation, their approaches still caused some defects that could cause performance degradation of carbon-based materials. Thus, effective strategies such as the optimization of functional materials and hybridization are required to obtain suitable materials for wearable sensors. In addition to the cost issues, it is necessary to further develop the high sensitivity and long operational stability of carbon-based sensor devices in order to realize practical applications that can precisely detect human activities or physiological signals.

Although textile-based sensors are more cost-effective compared to carbon-based materials, current textile-based sensors often require additional lamination or interlayer to fabricate sensors directly on fabric substrates. This is because the surface of textiles are usually rough, which can lead to weak adhesion, delamination of desired materials formed on textiles, and degradation of electrical performances. Due to the additional layers covering a substantial area of textiles, textile-based wearable sensors have lost the unique properties of textiles. Therefore, in order to realize a fully textile-based wearable sensor used in daily life, aforementioned materials, fabrication technologies, and various sensor devices must all be developed together. Furthermore, textile-based wearable sensors require excellent mechanical properties against various deformations such as bending, stretching, and twisting in order to be integrated into clothing for everyday life. In particular, the electrical performances of textile-based wearable sensors can be degraded during washing because of the mechanical deformations induced during the washing. Therefore, the durability of the washing process should be required to be further developed for use as wearable sensors in daily life.

In addition, interfacing textile-based sensors and electronics is still challenging. Since most silicon-based electronics are based on rigid or non-stretchable substrates, there is an issue of securing contacts between electronics and stretchable textile-based sensors. Creating active devices on a thread [133,134,135] or transistor-based active sensor arrays [159,160] could be potential solutions since the SNR of measured signal can be increased by on-thread or on-sensor amplifiers. These transistor-based active devices integrated on textile can also allow us to use various sensing parameters, such as current ratio, mobility, threshold voltage, etc., compared to a traditional capacitive or resistive-type devices. However, the power consumption of sensor devices should be considered for long-time continuous uses. Notably, textile-based energy harvesting or storage devices are required for these textile-based active devices, which is, however, beyond the scope of this review article.

Furthermore, for future routes with carbon- and textile-based wearable sensors in health monitoring, multifunctional sensing characteristics should not be overlooked. Although recent research on wearable devices exhibited great sensing performances such as high sensitivity, durability, and mechanical property, the function of discriminating multiple or intermixed stimuli is still limited. For the discrimination of intermixed stimuli, a lot of sensor units with different sensing functions are definitely required, which demands a new strategy to provide substantial advances for the state-of-the-art wearable device technology. Recently, Lee et al. reported a behavior-learned cross-reactive sensor array with artificial perception technology based on a machine learning process to detect and distinguish various intermixed stimuli. Unlike most previous wearable sensors focused on the configuration of sensor devices and nanostructured materials engineering technology, the pattern recognition and multimodal perception for intermixed stimuli are possible by adopting a machine learning algorithm [161]. The combination of carbon- and textile-based wearable sensor arrays with a machine learning algorithm could become a viable approach to realize practical wearable applications that can detect and discriminate multiple stimuli in health monitoring systems.

## 5. Conclusions

In this review, we have discussed the fabrication technologies and the wearable sensor devices for flexible substrate-based wearable sensors for health monitoring, which are mainly divided into two categories; (1) carbon-based sensors and (2) textile-based sensors. For demonstrating flexible and stretchable wearable sensors, the elements used in those sensors should be low-cost, reliable, easy, safe to use, multi-sensitive, and possible for long-term use. Considering the requirements, the approaches using carbon- and textile-based sensors would be the most suitable alternatives due to their outstanding advantages such as high mechanical and electrical characteristics. However, there are still numerous issues including high electrical performance, good stability, and an easy process to reach practical applications for health monitoring. In order to address these issues, innovative structural and material designs and novel processing technologies should be intensively researched and then introduced into wearable health monitoring sensors. Recently, most reported research for carbon- and textile-based approaches have contributed a detailed investigation of the high advanced wearable devices as well as their systems for health monitoring, resulting in the rapid development in process technology, new functional material/structure, and novel mechanism. We believe that advanced health-monitoring wearable sensor systems via newly designed technologies and materials for carbon/textile-based sensors can be a promising candidate for the new concept of next-generation wearable devices. Furthermore, with the introduction of the intellectualization and active matrix of wearable sensor arrays, a health monitoring system with carbon/textile-based sensors will become a new paradigm for diagnosing and treatment of medical conditions beyond the current clinical applications.

## Figures and Tables

**Table 1:** Summary of textile-based pressure/strain sensors and their sensing properties.

Textile	Sensing-Type	Configuration	Materials	Measurement Range	Sensitivity	Ref.
Warp-knitted Polyester Fabric	Pressure	3-Dimensional	Carbon black/Silicon elastomer composite	283 kPa	50.31 × 10^−3^ kPa^−1^	[139]
Silk Fabric	Pressure	3-Dimensional	Multi-layer structure with rGO	140 kPa	0.4 kPa^−1^	[136]
Knitted Polyester Fabric	Pressure	2-Dimensional	PEDOT:PSS/PDMS	0.25 N cm^−2^	No	[36]
Coaxial Fiber (Nylon/PU fiber)	Pressure	Cross contact	AgNW/PDMS-Carbon black	2.0 N	4.29 N^−1^	[137]
Nylon Fabric	Pressure	2-Dimensional	rGO	2500 kPa	5.2 Ω kPa^−1^	[140]
PVDF Nano Fiber	Pressure	2-Dimensional	PEDOT@PVDF	10 kPa	18.376 kPa^−1^	[138]
PU yarn	Strain	1-Dimensional	CB/CNC/NR composite	10%	39 (G.F)	[141]
Polyamide /Polyester Fibers	Strain	2-Dimensional	Carbon particles	30% (Angle: 120°)	No	[142]
Silk Fabric	Strain	2-Dimensional	Carbonization/Ecoflex	500%	37.5 (G.F)	[143]
Glass Fiber	Strain	1-Dimensional	Graphite Nano-platelet/epoxy	2.5%	17 (G.F)	[145]
Woven Fabric	Strain	2-Dimensional	Graphene (CVD)/PDMS	3%	223 (G.F)	[144]
PU and PE Fibers	Strain	1-Dimensional	rGO/PDMS	200%	35 (G.F)	[146]
